# COVID-19 in Brazil—Preliminary Analysis of Response Supported by Artificial Intelligence in Municipalities

**DOI:** 10.3389/fdgth.2021.648585

**Published:** 2021-06-17

**Authors:** Hugo M. P. Morales, Murilo Guedes, Jennifer S. Silva, Adriano Massuda

**Affiliations:** ^1^Department of Research, Instituto Laura Fressatto, Curitiba, Brazil; ^2^School of Medicine, Pontifícia Universidade Católica Do Paraná, Curitiba, Brazil; ^3^Department of Customer Success, Instituto Laura Fressatto, Curitiba, Brazil; ^4^Department of Administration, São Paulo School of Business Administration, Fundação Getulio Vargas, São Paulo, Brazil

**Keywords:** COVID-19, artificial intelligence, chatbot, public health, access, care coordination

## Abstract

The novel coronavirus disease (COVID-19) forced rapid adaptations in the way healthcare is delivered and coordinated by health systems. Brazil has a universal public health system (Sistema Unico de Saúde—SUS), being the main source of care for 75% of the population. Therefore, a saturation of the system was foreseen with the continuous increase of cases. The use of Artificial Intelligence (AI) to empower telehealth could help to tackle this by increasing a coordinated patient access to the health system. In the present study we describe a descriptive case report analyzing the use of Laura Digital Emergency Room—an AI-powered telehealth platform—in three different cities. It was computed around 130,000 interactions made by the chatbot and 24,162 patients completed the digital triage. Almost half (44.8%) of the patients were classified as having mild symptoms, 33.6% were classified as moderate and only 14.2% were classified as severe. The implementation of an AI-powered telehealth to increase accessibility while maintaining safety and leveraging value amid the unprecedent impact of the COVID-19 pandemic was feasible in Brazil and may reduce healthcare overload. New efforts to yield sustainability of affordable and scalable solutions are needed to truly leverage value in health care systems, particularly in the context of middle-low-income countries.

## Introduction

The novel coronavirus disease (COVID-19) seriously challenged health systems in the world. Emerging at the end of 2019 in Wuhan (China), the outbreak caused by the virus SARS-CoV-2 was declared as a pandemic by the World Health Organization (WHO) on March 11, 2020 (1). As 14 April 2021, the WHO COVID-19 situation report informed there have been over 137 million cases and over 3 million deaths since the start of the pandemic. With 354,617 deaths by the same date, Brazil ranks as the second most affected country in the world (1).

To face the Pandemic, countries developed different strategies. Lessons from previous coronavirus epidemics—SARS in 2003 and MERS in 2015—enabled governments in east and southeast Asia to take rapid and efficient health-systems responses to control SARS-CoV-2 transmission. In contrast, European and American countries which have the capacity to deal with an influenza epidemic were not prepared to face a coronavirus one. The late and disorganized response to the COVID-19 collapsed well-structured health systems and cost thousands of lives.

In this challenging context, innovative strategies have been described in order to support the COVID-19 response and the high pressure over the health system. Technological strategies played a pivotal role, especially in augmenting and organizing access to healthcare systems. Symptom checkers, which are self-directed risk assessments tools, has been shown to improve live quality and survival in oncology patients (2). Its use for COVID-19 is currently increasing and has been used to identify symptomatic patients or potential severe cases with the main objective to triage those individuals who would benefit from clinical assessment and/or management into further care (3). A recent French study showed that a national self-triage web application contributed to the alleviation of calls to the emergency calls centers and may be used for predicting increasing burden of hospital (4).

Another broadly adopted technology against COVID-19 are chatbots, which are Artificially intelligent (AI) based conversational agents. These applications can be accessed via a website or social media messaging platforms, such as WhatsApp and Facebook. Besides self-triage and personal risk-assessments, chatbot has been used to disseminate health information and knowledge, tracking COVID-19 symptoms and health aspects and monitor exposure and notifications (5). These novel technologies are excellent benefactors for facilitating progress in healthcare as they enable better accessibility and personalization for consumers and efficiency for healthcare providers and public health officials (6). Despite the increasing use, a recent review found only 9 studies, the majority of the studies coming from North America (5).

Brazil has a universal public health system (Sistema Unico de Saude—SUS), which is the main source of care for 75% of the population. Decentralized to the municipal level, health departments in the 5,570 municipalities largely handle the management of SUS, including co-financing, coordination of health programs, and delivery of health services. Despite the achievements in improving health outcomes since the implementation of SUS, deficiencies in its structure and governance persist, maintaining wide inequalities in distribution of health services across the country (7). These deficiencies have been exacerbated since fiscal austerity and health policy changes were implemented in 2016.

In the response to COVID-19, the absence of the federal government to assume its role of authority and leadership in combating the pandemic led municipal and state managers to build their own strategies to deal with the effects of the pandemic on their populations (8). Local responses varied in form, intensity, duration, and start and end times, to some extent associated with political alignments (9).

As a strategy to support health system management capacity at a municipal level, we developed a platform powered by Artificial Intelligence, called Laura Digital ER. By providing an innovative way for patients to have easy access to medical information via telehealth, the platform offers a solution to triage patients, to monitor the evolution of symptoms and to provide a credible and current source of information for patients. As a response to the pandemic, public-private partnerships, such as Inter-American Development Bank (IADB), BID Lab and Instituto Votorantim sponsored the implementation of the platform in municipal health systems.

Few studies have assessed the impact of deploying digital solutions at the municipal level. To fill this gap we describe three Brazilian municipality-level case reports setting out the early implementation of a digital triage and monitoring service which included the use of a chatbot utilizing algorithmic decision-making.

## Materials and Methods

### Study Design

This is an observational, descriptive study that aims to summarize the early experience of the deployment of a platform consisting on a decision-tree algorithm to perform COVID-19 risk assessment in patients seeking emergency room evaluation for acute symptoms amid the pandemic combined with a chatbot to both disseminate health informations and track patient's symptoms evolution when needed. The algorithm's primary goal was to ensure safety while increasing accessibility and decreasing healthcare usage of resources during the COVID-19 pandemic. The means by which the tool was assumed to deliver its value were through a Kanban framework, in which healthcare facilities could dynamically allocate resources according to demand. This descriptive study reports on data collected from July to October 2020 from three large municipalities in Brazil, namely Curitiba (almost 2 million inhabitants), São Bernardo do Campo (almost 900,000 inhabitants), and Catanduva (roughly 100,000 inhabitants).

### Algorithm Description

Employing a Natural Language Processing (NLP) framework, the Laura Digital ER platform was developed in April 2020 in response to the challenges created by the COVID-19 pandemic. Machine Learning algorithm was applied in order to ameliorate user experience in the process of answering basic questions and doubts about the pandemic (i.e., social distancing and preventive measures, such as how to wash hands, how and when to use masks). The chatbot was powered by RASA's NLP technology and the content was updated weekly by Laura's Team of health professionals, according to an analysis of the most commons question asked by the users. A powered chatbot was integrated into municipalities' websites to allow patient's interaction. Additionally, extensions to social media platforms and popular communication apps were ensured. The major objective of this chatbot is to provide the population with current and accurate information about COVID-19, including social distancing and preventive measures following CDC guidelines.

If a symptom was detected by the algorithm, the patient was redirected to a Triage Web Page in which a consent form was applied and, if signed, the individual was directed to a symptom checker. Once symptoms were verified, patients were classified into three main subgroups, following criteria defined by local authorities: mild, moderate or severe. Patients classified as mild disease were followed by a virtual non-human clinical pathway, in which every 72 h the algorithm interacted with individual through a messaging platform. Patients falling into moderate or severe disease categories were prompted to phone calls or teleconsultations with a healthcare provider. All patients, stratified by their severity, could be monitored by a healthcare provider in a platform allowed by the interface. In such a way, the process deployed through the algorithm ensured prioritization of individuals with more severe COVID-19 presentations while a continuous oversight by a healthcare provider was afforded, potentially allowing dynamic and optimal healthcare utilization using a Kanban framework.

### Ethical Aspects

This study follows the principles of the Declaration of Helsinki. All patients signed consent forms before data were extracted. Confidentiality and data privacy were ensured for all patients. The algorithm described here follows Brazilian local regulations for data protection n (Lei Geral de Proteção de Dados). Data were anonymized before any analyses were performed.

### Data Presentation

In this descriptive study, we report data on the algorithm utilization during a period during the COVID-19 pandemic in Brazil. Demographic data from the three cities in which the algorithm was deployed was extracted from the Brazilian Institute of Geography and Statistics (IBGE).

We calculated the number of patients who used the platform, the proportion of individuals stratified by COVID-19 severity as assessed by the algorithm, the absolute number of patient interactions with the algorithm and number of patients exclusively followed by non-human interactions from July to October 2020.

## Results

The three cities in which the algorithm was deployed were Curitiba, São Bernardo do Campo, and Catanduva (Table 1). All these regions already had a call center organized to allow triage of patients with COVID-19 symptoms, in which healthcare providers who were not able to work in the frontline (e.g., elder of with comorbidities) were relocated to work in such a system. These healthcare providers were responsible to assess and determine workflow for patients with COVID-19 symptoms, such as evaluating potential severity and recommending in-person visits in the emergency room. Over the course of the COVID-19 pandemic, the total amount of calls gradually increased, with up to 1,000 calls per day in some of these regions. This rapid increase raised concerns regarding potential burden and lack of adequate follow-up for individuals with varying risks of time, as reflected in COVID-19 natural history in which more severe features ensue around 7–10 days. In this framework, healthcare works called individuals 3–5 times per day over a 14-day period of follow-up.

**Table 1:** Stratification of alert severity per municipality.

**Metric**	**Curitiba**	**Catanduva**	**Sao Bernardo do Campo**	**Total**
City population	1,948,626	122,497	838,936	2,910,059
Chatbot interactions	96,626	31,732	9,420	133,778
Persons who interacted with the platform	14,646	6,262	1,510	22,418
**Total alerts**	17,498	5,903	761	24,162
Gray (asymptomatic)	1,406	258	92	1,756
Blue (mild)	7,077	3,185	345	10,607
Yellow (moderate)	5,637	2,092	219	7,948
Red (severe)	2,880	369	105	3,353

With the deployment of the AI algorithm in these cities, the workflow was changed to ensure greater patient interaction with the healthcare system by reducing the total amount of time spent by healthcare professionals in low-risk patients, such as those with non-severe COVID-19 presentations. During the period described in this study (July to October 2020), the NLP-powered chatbot interacted with 22,418 persons, performing more than 133,700 interactions (i.e., answered questions). Most common questions answered by the chatbot were related to COVID-19 test costs and locations to perform it, issues relating to transmission after close contact with a SARS-CoV2 infected individual and the average time to recover from COVID-19.

**Figure 1:**
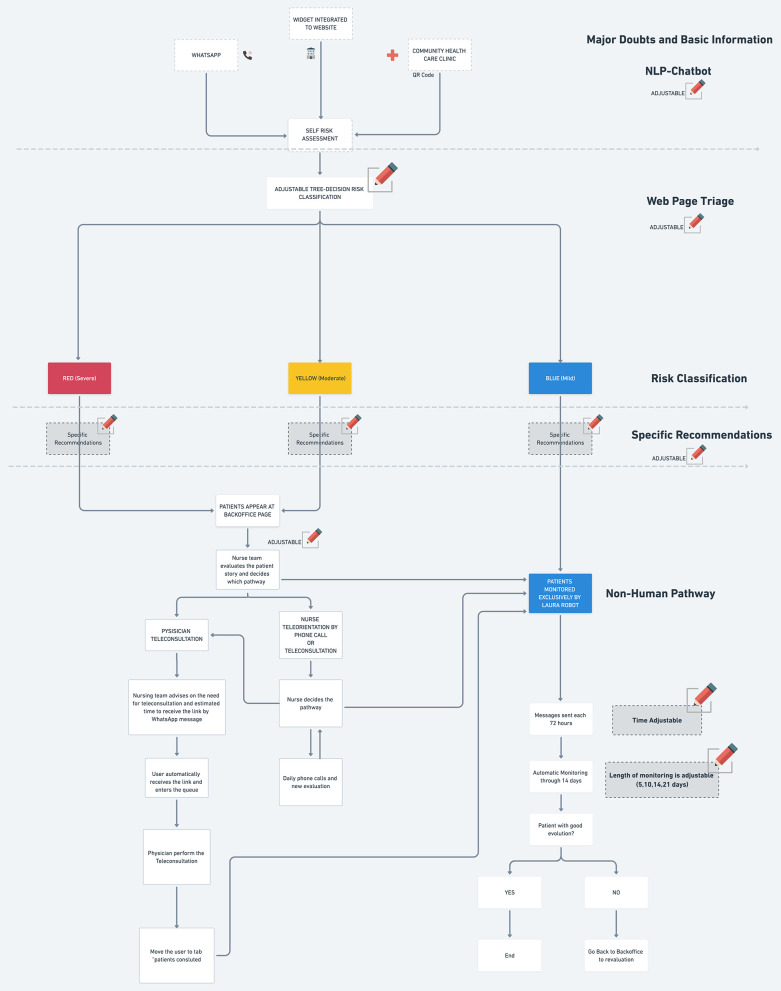
AI-chatbot for risk COVID-19 severity risk assessment. The flow-chart describes all steps in the interaction between patients and the algorithm, as well as the indicated interventions according to the COVID-19 severity based on symptoms assessment evaluation by the technology.

Risk assessment was categorized in four domains: gray (asymptomatic patient), blue (mild symptoms), yellow (moderate symptoms), and red (severe symptoms) based on interactions with health departments in each city. From the 24,162 interactions resulting in symptom evaluating by the NLP-algorithm, up to 45% were classified as mild disease and 14% as severe (Figure 1). Complete distribution of severity assessment is described in Table 1.

In terms of patient population, most individuals were older than 60 years old. The most commonly reported symptoms were cough (55%), sore throat (42.8%), and headache (39.8%). Symptom distribution is summarized in Table 2, stratified per location.

**Table 2:** Distribution of the most reported symptoms.

**Symptom**	**Curitiba**	**Catanduva**	**Sao Bernardo do Campo**	**Total**	
**Total**	**19,390**	**9,090**	**907**	**29,387**	
Cough *n* (%)	4,381 (22.6)	3,216 (35.4)	263 (29)	7,860	26.7%
Sore throat *n* (%)	4,304 (22.2)	2,452 (27)	238 (26.2)	6,994	23.8%
Headache *n* (%)	4,593 (23.7)	2,276 (25)	177 (19.5)	7,046	24.0%
Myalgia *n* (%)	3,122 (16.1)	1,913 (21)	196 (21.6)	5,231	17.8%
Fever *n* (%)	3,471 (17.9)	2,048 (22.5)	156 (17.2)	5,675	19.3%
Anosmia *n* (%)	2,244 (11.6)	1,009 (11.1)	95 (10.5)	3,348	11.4%
Diarrhea *n* (%)	1,569 (8.1)	627 (6.9)	49 (5.4)	2,245	7.6%
Dyspneia *n* (%)	1,412 (7.3)	1,497 (16.5)	88 (9.7)	2,997	10.2%

*One patient can have more than one symptom. n = 24,162*.

## Discussion

In this descriptive study, we aimed to summarize the initial phase of the first initiative in Brazil to provide a scalable tool to ensure better healthcare access to individuals while ensuring safety and contributing to better operations amid the COVID-19 pandemic. As far as we know, this is the first description of a telemedicine platform powered by an AI-chatbot to tackle COVID-19 issues as a decentralized and adjustable tool in Brazilian municipalities.

In the present study, almost half of patients who answered the symptoms checker were classified as having mild symptoms, therefore they could be safely followed without an in-person visit to the emergency room, as defined in COVID-19 management guidelines. Importantly, these patients classified as mildly symptomatic remained on the non-human clinical pathway, having their clinical status assessed by direct conversation with the chatbot. Since it is established a policy of making 3–4 phone calls to check the clinical evolution of these mild cases, we can assume that there was a saving of more than 60,000 phone calls after the algorithm's implementation. Therefore, even in this initial description, the technology is thought to have optimized human resources to more demanding areas in a severely overloaded system.

The sustainability of implementing such innovative tools in a country, such as Brazil requires several structural and cultural adaptations, as highlighted in this study (5). The algorithm was conceived to be used in the public health system in Brazil, leveraging available resources in specific regions. The three regions we describe in this study belong to a group of a relatively higher economic status in Brazil, in which technology use is broader and thereby patients can have better accessibility to technological solutions. The complexity of a continental country, such as Brazil implies the widespread adoption of innovative solutions in healthcare will need to address many challenges (10). Other than technological resources, sustainability in terms of financial structures for such projects are key limiting points to unleash innovative solutions (10). For the initiative presented here, a public and private collaborative effort was leveraged to implement a potential solution for better resource allocation amid the COVID-19 pandemic, as described. Continuing efforts to allow affordability in the processes of implementation of new, innovative tools in public-private partnerships are necessary in order to guarantee a stable environment in which continuous quality improvement can be developed. In a country in which the majority of individuals are covered by a public health system, sustainable innovation to allow wider access and to lever equity in healthcare implies coordinated efforts between multiple stakeholders. The coordination between these stakeholders needs to ensure a well-designed implementation plan, as well as rigorous metrics for performance and safety evaluation over time, which can be challenging in a system without a unified medical record (10–12). Indeed, in the public system in Brazil, the lack of a centralized medical record to allow follow-up of individuals who interacted with the algorithm has been a main limitation so far in terms of establishing outcomes related to technological interventions, such as the one described in this paper.

The total number of symptom checker evaluations was considered relatively low taking into account the total population assessed and it varied widely among the cities. The municipality of Catanduva (5,111 people who used the tool/100,000 inhabitants) presented higher usage rates in comparison to Curitiba (751/100,000 inhabitants) and São Bernardo do Campo (179/100,00 inhabitants). A possible explanation is the municipality's strong commitment to the platform in organizational and, mainly, marketing terms. Sao Bernardo do Campo, which have the lowest usage in the studied period, implemented a strong advertising campaign to use the tool with great results. During the months of October, November, and December (up to the 30th), there were 41,406 persons who interacted with the chatbot and more than 2,500 patients completed the self-risk assessment. The lack of national guidelines for the adoption of digital solutions in healthcare could be one of the main factors for this disparity because the project prioritization was totally dependent on each city's commitment. The Ministry of Health have implemented a centralized chatbot solution for COVID-19 but unfortunately it was not accompanied by national guidelines and there was no possibility of customization or adapting to the existing local workflow of the cities. We believe that a national plan for adopting digital healthcare is essential to transformation in healthcare toward leveraging optimal value to all stakeholders, but essentially and more importantly, to the end users, whose outcomes are at the core of all endeavors.

This study has several limitations. We provided a descriptive study on the early experience of the implementation of an AI-based solution to improve health care utilization amid the COVID-19 pandemic in the public health system in Brazil. We were unable to report health outcomes associated to the platform, due to the early stage of the report and because of the lack of uniform and centralized medical records data in the country. Additionally, although our symptom check was intuitive and validated in interactions with medical directors and healthcare leaders in each municipality in Brazil, our questionnaires were not previously validated or standardized.

Notably, this early report has several strengths. In spite of all limitations, we were able to describe a successful implementation of an AI-powered chatbot to assess COVID-19 related risks and to provide optimal healthcare utilization through flexible, on-demand and accessible structures. These findings corroborate with others from Austria, Switzerland and the USA, suggesting that the use of this technology may decrease the healthcare system overload and increase access to healthcare system (13–15). All efforts summarized here were validated along with health authorities in the country, therefore safety was ensured based on rigorous evaluation by state departments in each city where the algorithm was implemented. Therefore, we provide the first description of a successful algorithm to improve healthcare utilization amid the COVID-19 pandemic, leveraging value while minimizing burden during unprecedent times in a heterogeneous and widely affected country as Brazil. This report highlights that well-structured public-private initiatives, coupled with innovative endeavors and sustainable incentives, yields value in healthcare even among low-middle income countries. Technology and telehealth can leverage existing structures toward greater healthcare management, improving accessibility through safe and optimal solutions.

## Conclusions

The development and deployment of an AI powered telehealth aiming for greater access and optimizing care coordination is feasible, advisable, and may reduce healthcare overload. In order to succeed, the platform should be adaptable to local needs, including being capable of changing the decision-tree algorithm. A national policy for digital healthcare transformation could guide and enhance adoption of innovative technologies at municipalities-levels.

## Data Availability Statement

The original contributions presented in the study are included in the article/supplementary material, further inquiries can be directed to the corresponding author/s.

## Author Contributions

HM and AM conceived the paper. JSS collected data. All authors made substantial contributions in writing the paper and contributed to the article and approved the submitted version.

## Conflict of Interest

HM, JS, and MG are employees of Laura, the company that developed Laura Digital ER. The remaining author declares that the research was conducted in the absence of any commercial or financial relationships that could be construed as a potential conflict of interest.
